# Association of *APOA5 rs2075291* and *CIDEB rs2144492* polymorphisms with hypertriglyceridemia in individuals with traditional Chinese medicine dampness syndrome: a case-control study

**DOI:** 10.3389/fgene.2025.1654501

**Published:** 2025-10-17

**Authors:** Na Liu, Hongli Zeng, Xiangsheng Cai, Shuo Yang, Xinyan Chen, Guli Jiang, Jiamin Yuan, Jianxiong Cai, Hui Zhou

**Affiliations:** ^1^ Guangzhou 11th People’s Hospital, Guangzhou Cadre and Talent Health Management Centre, Guangzhou, China; ^2^ State Key Laboratory of Dampness Syndrome of Chinese Medicine, The Second Affiliated Hospital of Guangzhou University of Chinese Medicine, Guangzhou, China; ^3^ Guangdong Provincial Key Laboratory of Clinical Research on Traditional Chinese Medicine Syndrome, The Second Affiliated Hospital of Guangzhou University of Chinese Medicine, Guangzhou, China

**Keywords:** hypertriglyceridemia, dampness syndrome, APOA5 gene, CIDEB gene, singlenucleotide polymorphisms

## Abstract

**Purpose:**

To investigate the association between polymorphisms of the *APOA5 rs2075291* and *CIDEB rs2144492* loci and hypertriglyceridemia (HTG) in a population with Traditional Chinese Medicine (TCM) dampness syndrome.

**Methods:**

A case-control study was conducted, enrolling 100 HTG patients and 100 age-matched controls with normal triglyceride levels from the physical examination cohort at Guangzhou 11th People’s Hospital (January–December 2023)*.* Peripheral blood samples were collected to analyze *APOA5 rs2075291* and *CIDEB rs2144492* polymorphisms using PCR and sequencing. Lipid profiles were measured via an automated biochemical analyzer. Statistical analyses (chi-square tests, correlation analysis, and logistic regression) evaluated associations among gene polymorphisms, dampness syndrome, and HTG.

**Results:**

The observation group showed significant differences in genotype frequencies of *APOA5 rs2075291* (OR = 2.916, 95% CI:1.160–7.334, χ^2^ *p* = 0.019) and *CIDEB rs2144492* (OR = 1.688, 95% CI:0.886–3.141, χ^2^ *p* = 0.042) versus the control group. Significant intergroup differences were also observed in allele frequencies of *APOA5 rs2075291* (OR = 2.727, 95% CI:1.113–6.682, χ^2^ *p* = 0.023) and *CIDEB rs2144492* (OR = 1.837, 95% CI:1.040–3.244, χ^2^ *p* = 0.034). Stratified by dampness syndrome status, in the dampness syndrome subgroup, the HTG group had a higher frequency of *CIDEB rs2144492* TG/TT genotypes than controls, though the difference was not significant (OR = 2.065, 95% CI:0.816–5.226, χ^2^ *p* = 0.146). No significant difference in gene frequency was observed after FDR correction (*p* = 0.043, FDR threshold = 0.042). *APOA5 rs2075291* showed no significant genotype/allele frequency differences (*p* > 0.05). In the non-dampness subgroup, FDR correction (*p* ≤ 0.033) revealed no significant differences in *APOA5 rs2075291* genotype (OR = 4.083, 95% CI:0.977–17.063, χ^2^ *p* = 0.041) or allele frequencies (*p* = 0.05), nor in CIDEB rs2144492 genotypes/allele frequencies (*p* > 0.05). Triglyceride levels did not differ significantly between dampness/non-dampness groups across genotypes (*p* > 0.05). Multivariate logistic regression identified male gender, higher BMI, dampness syndrome, and *APOA5 rs2075291* genotype as independent risk factors for HTG (*p* < 0.05), while *CIDEB rs2144492* trended toward significance (*p* = 0.05).

**Conclusion:**

*APOA5 rs2075291* and *CIDEB rs2144492* polymorphisms are associated with hypertriglyceridemia. Dampness syndrome individuals with C*IDEB rs2144492* variants may have increased HTG predisposition. Larger cohort studies are warranted to validate these findings and explore underlying mechanisms.

## Introduction

Hypertriglyceridemia is a common lipid metabolism disorder and well-established risk factor for cardiovascular diseases (CVDs), including myocardial infarction and ischemic stroke (Xie, 2023; Jørgensen et al., 2013; Freiberg et al., 2008; Nordestgaard and Varbo, 2014). The etiology of HTG involves a complex interplay of genetic and environmental factors, with single nucleotide polymorphisms (SNPs) in genes such as *APOA5* and *CIDEB* playing pivotal roles in triglyceride regulation (Xu Yn and Pan, 2022; Steinhagen-Thiessen et al., 2017; Kypreos and Zannis, 2006; Guardiola and Ribalta, 2017; Xu et al., 2012).

*APOA5* encodes a 366-amino acid protein found in triglyceride-rich lipoproteins and high-density lipoprotein (HDL) particles (Zafar et al., 2019; Srivastava et al., 2015; Su et al., 2018). It is an effective regulator of plasma triglyceride (TG) and HDL cholesterol (HDL-C) levels (Ajjemami et al., 2015), and genetic variants in *APOA5* are strong predictors of hypertriglyceridemia-related cardiovascular risk (Ding et al., 2012). Among *APOA5* pathogenic mutations, the *rs2075291* (Gly185Cys) variant is the most prevalent (Liu et al., 2024). A study (An et al., 2011) involving 406 Uyghur and 527 Han healthy physical examinees in Xinjiang, China, found that the distribution frequencies of the three genotypes of *ApoA5* gene *rs2075291* in the Uyghur group were 93.1% for the GG type, 6.7% for the GT type, and 0.25% for the TT type; those in the Han group were 90.7% for the GG type, 9.3% for the GT type, and no TT type. There was no statistically significant difference in the genotypic distribution between the two groups.

The *CIDE* family includes *CIDEA*, *CIDEB* and Fsp27 (*CIDEC* in humans) (Zhang et al., 2014), which were initially implicated in mammalian apoptosis (Park, 2015). However, subsequent research has revealed that *CIDE* proteins are critical regulators of multiple lipid metabolic pathways and lipid homeostasis (Lajnaf et al., 2023). *CIDEB*, an endoplasmic reticulum and lipid droplet-associated protein, located on human chromosome 14q11 (Ping et al., 2022), is involved in regulating lipid metabolism and related disorders (Xu et al., 2016). Recent studies have demonstrated that *CIDEB* promotes fatty acid synthesis, adipocyte formation, and hepatic triglyceride synthesis and storage (Li et al., 2010; Ng et al., 2021). A study (Liu and Zhan, 2016) on 528 Han Chinese individuals in Henan, China, found that the *CIDEB rs2144492* locus is associated with TG. The *CIDEB* gene polymorphism and the ATCC haplotype of the *CIDEB* gene play a certain role in the risk of HTG.

In traditional Chinese medicine, dampness syndrome stems from impaired body fluid metabolism, presenting with symptoms like fatigue, abdominal distension, and a slippery tongue coating (Zhu Wf, 2011). Epidemiological evidence indicates that populations in humid regions (e.g., Lingnan) exhibit elevated triglyceride levels, which may be associated with dampness syndrome (Chen and Huang, 2022; Zhang Bc, 2020; Zhou et al., 2024). However, the link between TCM dampness syndrome and HTG-related genetic polymorphisms remains unclear. This study investigates *APOA5 rs2075291* and *CIDEB rs2144492* polymorphisms in HTG patients with dampness syndrome, exploring the combined impact of genetic and TCM-specific factors on lipid metabolism.

## Participants and methods

### Subjects

A total of 200 participants (100 HTG cases and 100 controls) were recruited from the physical examination cohort at Guangzhou 11th People’s Hospital between January and December 2023. To control for population stratification, participants were proportionally matched between the observation and control groups. Only Han Chinese individuals were included, excluding other ethnic groups to avoid genetic heterogeneity confounding the results. All participants completed the TCM Dampness Syndrome Assessment Scale. Ages ranged from 18 to 75 years (mean: 47.15 ± 11.93), with 134 males (67%; mean age 47.42 ± 10.63) and 66 females (33%; mean age 46.61 ± 14.29). This study was approved by the Ethics Committee of Guangzhou Cadre Health Management Centre(Ethics Number: JGZX-2023-06), and written informed consent was obtained from all participants.

### Diagnostic criteria

1.HTG diagnosis (Wang ZW, 2024): Fasting TG ≥ 1.7 mmol/L(150 mg/dL). Controls: Total cholesterol (TC) < 5.2 mmol/L(200 mg/dL), low-density lipoprotein cholesterol (LDL-C) < 3.4 mmol/L (130 mg/dL), HDL-C≥ 1.0 mmol/L(40 mg/dL) and TG < 1.70 mmol/L(150 mg/dL). 2.Dampness syndrome diagnosis (Lu Ty and Cai, 2021): Evaluated using the TCM Dampness Syndrome Diagnostic and Evaluation Scale (National Key Laboratory of TCM Dampness Syndrome, Ministry-Province Co-Constructed). This 30-item self-assessment scale (total score 120 points) defines: No dampness syndrome 0–19 points; Dampness syndrome ≥20 points.

### Inclusion and exclusion criteria

The inclusion criteria were as follows: ① completed the TCM Dampness Syndrome Evaluation Scale and obtained a score greater than or equal to 0; ② was able to provide written informed consent, cooperate with the completion of the questionnaire and provide a blood sample; ③ aged ≥20 years old and ≤75 years old. The exclusion criteria were as follows: ① Inability to cooperate with the study; ② History of mental disorders; ③ Pregnant or lactating women; ④ Patients with diabetes, hypothyroidism, nephrotic syndrome, liver/kidney diseases, heavy alcohol consumption, or those taking lipid-altering medications (statins/fibrates/omega-3 fatty acids, retinoids, steroids, beta-blockers, antiretrovirals).

### Methods and data collection

Demographic, clinical, and biochemical data were retrieved from hospital records. Genotyping was performed via PCR and Sanger sequencing. All investigators specialized in TCM or integrated Chinese-Western medicine and were trained in the study’s standard operating procedures. Participants were randomly selected from outpatient attendees, with trained investigators assisting in questionnaire completion to ensure data integrity and reduce bias. Questionnaire components: ① General demographics (age, gender, etc.); ② Medical history; ③ TCM Dampness Syndrome Assessment Scale. Physical examinations: Height, weight, body mass index (BMI), blood pressure, waist circumference (WC), etc. Laboratory assessments: ① Biochemical markers: TC, TG, LDL-C, HDL-C, apolipoprotein AI, apolipoprotein B; ② Genetic polymorphisms: *APOA5 rs2075291* and *CIDEB rs2144492* loci. Genomic DNA extraction, PCR amplification, and sequencing were conducted by Guangzhou Aiji Biotechnology Co., Ltd.

Primer sequences: For *ApoA5 rs2075291*: Forward primer: 5′-CAG​CAA​CTG​AAG​CCC​TAC​ACG-3′, Reverse primer: 5′-ATG​CCG​CTC​ACC​AGC​TCT​CG-3′, Product length: 227 bp.

For *CIDEB rs2144492*: Forward primer: 5′-CTT​ATG​GCT​TCT​CCA​GTA​GGT-3′, Reverse primer: 5′-GTA​TGT​GTG​TCT​TTG​GTG​ATG​A-3′, Product length: 194 bp.

PCR reaction conditions: Initial denaturation at 94 C for 5 min; Denaturation at 94 C for 30 s; Annealing at 56 C for 30 s; Extension at 72 C for 30 s; 35 cycles in total; Final extension at 72 C for 5 min after the last cycle; Storage at 4 C.

The amplification products were analyzed by 1.5% agarose gel electrophoresis. The genotyping success rate and repeat concordance rate for *APOA5 rs2075291* and *CIDEB rs2144492* SNPs both reached 100%, satisfying quality control criteria.

### Statistical analysis

Data were analyzed using SPSS 26.0. Measurement data were expressed as mean ± standard deviation. Subgroup comparisons were performed via t-tests or analysis of variance. Pearson correlation analysis was applied for normally distributed data, while Spearman correlation was used for non-normally distributed data. Allele and genotype frequencies (calculated via genotype counting) were compared using chi-square tests or Fisher’s exact test. The false discovery rate (FDR) was corrected via the Benjamini–Hochberg method, with corrected significant results reported. The Hardy-Weinberg equilibrium (HWE) was assessed for polymorphic locus genotype distributions. The additive model served as the primary model, with dominant/recessive models as secondary. Genotypes were coded as 0/1/2 under the additive model. Models reported the Area Under the Curve (AUC), Hosmer-Lemeshow test (HL) *p*-value, and maximum variance inflation factor (VIF), including *APOA5*×dampness and *CIDEB* × dampness interaction terms. Post-hoc power analysis was conducted using the expected minor allele frequency (MAF) and observed OR values. Binary logistic regression was used to identify factors associated with HTG, with *p* < 0.05 denoting statistical significance.

## Results

### Comparison of baseline characteristics between hypertriglyceridemia group and control group

As shown in Table 1, the HTG group had significantly higher waist circumference, BMI, TG, TC, LDL-C, apolipoprotein B, and dampness syndrome scores compared to controls (*p* < 0.05). Conversely, HDL-C and apolipoprotein AI were lower in the HTG group (*p* < 0.05). There was no significant age difference between groups (*p* = 0.152). Collinearity assessment for BMI and waist circumference showed a VIF of 1.

**TABLE 1:** Comparison of baseline characteristics between hypertriglyceridemia group and control group.

Variable	HTG group,n = 100	Control group,n = 100	t/x^2^	*P* Value
Age (years)	48.36 ± 9.56	45.94 ± 13.85	−1.44	0.152
Gender (Male/Female)	84/16	50/50	26.14	0.000
WC (cm)	88.14 ± 7.45	79.77 ± 10.02	−6.70	0.000
BMI(kg/m^2^)	26.17 ± 2.62	23.71 ± 3.42	−5.70	0.000
TG(mmol/L)	3.08 ± 1.93	1.01 ± 0.34	−10.55	0.000
TC(mmol/L)	5.35 ± 0.84	4.98 ± 0.82	−3.15	0.002
HDL-C(mmol/L)	1.22 ± 0.23	1.58 ± 0.33	8.90	0.000
LDL-C(mmol/L)	2.95 ± 0.86	2.82 ± 0.71	−1.17	0.245
apolipoprotein AI	1.33 ± 0.23	1.50 ± 0.27	4.77	0.000
apolipoproteinB	1.11 ± 0.23	0.94 ± 0.22	−5.46	0.000
Dampness Syndrome Score	29.02 ± 18.44	23.10 ± 14.82	−2.50	0.013

HTG = hypertriglyceridemia; WC = waist circumference; BMI = body mass index; TG = triglyceride; TC = total cholesterol; LDL-C = low-density lipoprotein cholesterol; HDL-C = high-density lipoprotein cholesterol.

### *APOA5 rs2075291* genotype and allele frequency in different groups

The polymorphic genotypes of the *APOA5 rs2075291* locus in both groups were in Hardy-Weinberg equilibrium (*p* > 0.05). An interaction was observed between *APOA5 rs2075291* and dampness syndrome in the overall population (F = 5.796, *p* = 0.004). As presented in Table 2, after FDR correction (*p* ≤ 0.033), the genotype of *APOA5 rs2075291* differed significantly between the HTG and control groups (OR = 2.916, 95% CI:1.160–7.334, χ^2^ *p* = 0.019), with significant intergroup differences in allele frequencies (OR = 2.727, 95% CI:1.113–6.682, χ^2^ *p* = 0.023). In the dampness syndrome subgroup, neither genotype (OR = 2.241, 95% CI:0.667–7.527, χ^2^ *p* = 0.183) nor allele frequencies (*p* = 0.200) showed significant differences. In the non-dampness syndrome subgroup, the HTG group showed no significant difference from the control group in *APOA5 rs2075291* genotype frequency (OR = 4.083, 95% CI:0.977–17.063, χ^2^ *p* = 0.041) or allele frequencies (*p* = 0.05).

**TABLE 2:** *APOA5* rs2075291 Genotype and Allele Frequency in different groups [n(%)].

Group	Total n	GG genotype	GT genotype	G allele	T Allele
In the entire research population					
HTG group	100	82 (82%)	18 (18%)	182 (91.0%)	18 (9.0%)
Control group	100	93 (93%)	7 (7%)	193 (96.5%)	7 (3.5%)
χ^2^		5.531		5.163	
*P*		0.019		0.023	
In People with dampness syndrome					
HTG group	65	54 (83.08%)	11 (16.92%)	119 (91.5%)	11 (8.5%)
Control group	48	44 (91.67%)	4 (8.33%)	92 (95.8%)	4 (4.2%)
χ^2^		1.77		1.644	
*P*		0.183		0.200	
In People without dampness syndrome					
HTG group	35	28 (80%)	7 (20%)	63 (90.0%)	7 (10.0%)
Control group	52	49 (94.2%)	3 (5.80%)	101 (97.1%)	3 (2.9%)
χ^2^	4.164			3.844	
P	0.041			0.050	

In the entire research population, the HWE p-values were 0.261 for the HTG, group and 0.744 for the control group. In People with dampness syndrome, the HWE p-values were 0.731 for the HTG, group and 0.723 for the control group. In People without dampness syndrome, the HWE p-values were 0.281 for the HTG, group and 0.783 for the control group.

### *CIDEB rs2144492* genotype and allele frequency in different groups

The genotype distribution of the *CIDEB rs2144492* locus polymorphism in both groups was in Hardy-Weinberg equilibrium (P > 0.05). An interaction between *CIDEB rs2144492* and dampness syndrome was observed in the overall population (F = 5.796, p = 0.004). As shown in Table 3, after FDR correction (*p* ≤ 0.042), Fisher’s exact test showed significant differences in *CIDE-B rs2144492* genotype (OR = 1.688, 95% CI:0.886–3.141, χ^2^ *p* = 0.042) and allele frequency (OR = 1.837, 95% CI:1.040–3.244, χ^2^ *p* = 0.034) between HTG and control groups. In the dampness syndrome subgroup, the HTG group exhibited a higher frequency of CIDEB rs2144492 TG/TT genotypes, though the difference did not reach statistical significance (OR = 2.065, 95% CI:0.816–5.226, χ^2^ *p* = 0.146). Similarly, no significant difference was observed in allele frequencies between the two groups (*p* = 0.043). In the non-dampness subgroup, neither *CIDEB rs2144492* genotype (OR = 1.604, 95% CI:0.639–4.023, χ^2^ *p* = 0.281) nor allele frequency (*p* = 0.250) differed significantly between groups.

**TABLE 3:** *CIDEB rs2144492* Genotype and Allele Frequency in different groups [n(%)].

Group	Total n	GG genotype	TG genotype	TT^a^ genotype	G allele	T Allele
In the entire research population						
HTG group	100	68 (68%)	27 (27%)	5 (5%)	163 (81.5%)	37 (18.5%)
Control group	100	78 (78%)	22 (22%)	0 (0%)	178 (89%)	22 (11%)
χ^2^		6.063			4.473	
*P*		0.042			0.034	
In People with dampness syndrome						
HTG group	65	46 (70.8%)	15 (23.1%)	4 (6.2%)	107 (82.3%)	23 (17.7%)
Control group	48	40 (83.3%)	8 (16.7%)	0 (0%)	88 (91.7%)	8 (8.3%)
χ^2^		3.716			4.087	
*P*		0.146			0.043	
In People without dampness syndrome						
HTG group	35	22(62.9%)	12 (34.3%)	1 (2.9%)	56 (80%)	14 (20%)
Control group	52	38(73.1%)	14 (26.9%)	0 (0%)	90 (86.5%)	14 (13.5%)
χ^2^		2.124			1.325	
*P*		0.281			0.250	

In the entire research population, the HWE p-values were 0.435 for the HTG, group and 0.271 for the control group. In People with dampness syndrome, the HWE p-values were 0.087 for the HTG, group and 0.680 for the control group. In People without dampness syndrome, the HWE p-values were 0.673 for the HTG, group and 0.262 for the control group.

a: Fisher’s exact test.

### Comparison of mean triglyceride levels among different genotypes in dampness syndrome and non-dampness syndrome populations

Using log-transformed mean TG as the dependent variable, interactions of *APOA5*×dampness syndrome and *CIDEB* × dampness syndrome were tested. Interaction *p*-values were: *APOA5*×dampness syndrome (*p* = 0.322) and *CIDEB* × dampness syndrome (*p* = 0.966). As shown in Table 4, mean triglyceride levels did not differ significantly between dampness and non-dampness groups for *APOA5 rs2075291* GG/GT genotypes or *CIDEB rs2144492* GG/TG/TT genotypes (*p* > 0.05). An ANCOVA model for log-transformed TG was fitted, adjusting for age, gender, BMI, dampness syndrome, and genotype. Adjusted mean differences are reported in Table 4.

**TABLE 4:** Comparison of mean triglyceride levels among different genotypes in dampness syndrome and non-dampness syndrome populations.

Group	n	TG (mmol/L)	t	P	Adjusted mean differences (%)
*APOA5* GG genotype - Dampness	98	1.64 ± 1.98	−1.608	0.110	17.73
*APOA5* GG genotype - Non-Dampness	77	1.41 ± 1.79			
*APOA5* GT genotype - Dampness	15	2.31 ± 1.85	−0.044	0.966	1.32
*APOA5* GT genotype - Non-Dampness	10	2.28 ± 2.61			
*CIDEB* GG genotype - Dampness	86	1.69 ± 1.92	−1.825	0.070	21.58
*CIDEB* GG genotype - Non-dampness syndrome	60	1.39 ± 1.86			
*CIDEB* TG genotype - Dampness syndrome	23	1.67 ± 2.25	0.009	0.993	0.24
*CIDEB* TG genotype - Non-dampness syndrome	26	1.67 ± 1.97			
*CIDEB* TT genotype - Dampness syndrome	4	2.86 ± 1.25	0.809	0.478	22.45
*CIDEB* TT genotype - Non-dampness syndrome	1	3.49			

TG = triglyceride.

### Point-biserial correlation analysis of hypertriglyceridemia and related indicators in different groups

As shown in Table 5, in the overall population, HTG showed significant correlations with age, gender, body mass index, *APOA5 rs2075291* genotype, and dampness syndrome (*p* < 0.05). In the dampness syndrome subgroup, HTG was significantly associated with gender and body mass index, whereas in the non-dampness syndrome subgroup, it was significantly correlated with gender, body mass index, and *APOA5 rs2075291* genotype (*p* < 0.05 for all).

**TABLE 5:** Point-biserial Correlation Analysis of Hypertriglyceridemia and Related Indicators in different groups.

Variable	Totality		Dampness syndrome group		Non-dampness syndrome group	
	*R*	*P*	*R*	*P*	*R*	*P*
Age	0.150	0.034	0.175	0.063	0.159	0.141
Gender	0.362	<0.001	0.450	<0.001	0.250	0.020
BMI	0.384	<0.001	0.425	<0.001	0.266	0.013
*APOA5* rs2075291 genotype	0.166	0.019	0.125	0.187	0.219	0.042
*CIDEB* rs2144492 genotype	0.124	0.079	0.157	0.097	0.116	0.284
Dampness Syndrome	0.171	0.015				

HTG = hypertriglyceridemia; Assignment: Gender (Male = 1, Female = 0); *APOA5* rs2075291 genotype (G/G = 0, G/T = 1,T/T genotype not detected); *CIDEB*, rs2144492 genotype (G/G = 0,T/G = 1,T/T = 2).Note: Dampness Syndrome was the grouping variable and no intra-group correlation analysis was conducted.

### Binary logistic regression analysis of hypertriglyceridemia and related indicators

Variables were selected based on correlation analysis results and primary research objectives, with genotypes encoded additively. Binary multivariate logistic regression was performed, using HTG status (presence/absence) as the dependent variable and including dampness syndrome, *APOA5 rs2075291* genotype (GG = 0, GT = 1), and *CIDE-B rs2144492* genotype (GG = 0, TG = 1, TT = 2) as independent predictors. Collinearity assessment showed all tolerance values > 0.1 and max VIF = 1.310, indicating no multicollinearity. AUC values for variables (age = 0.586, gender = 0.670, *APOA5* = 0.555, *CIDEB* = 0.556, BMI = 0.721, dampness syndrome = 0.585) all exceeded 0.5. The Hosmer-Lemeshow test (χ^2^ = 11.884, *p* = 0.156) indicated good model fit. Logistic regression identified male gender, higher BMI, dampness syndrome, and *APOA5 rs2075291* genotype as independent HTG risk factors (*p* < 0.05), while *CIDEB rs2144492* genotype trended toward significance (*p* = 0.05), as shown in Table 6.

**TABLE 6:** Binary logistic regression analysis of hypertriglyceridemia and related indicators.

Indicator	β	Wald	Significance	Exp(B)	95%Confidence interval
Age	0.018	1.649	0.199	1.018	0.991–1.046
Gender(Female = 0,Male = 1)	1.161	9.171	0.002	3.194	1.506–6.774
BMI	0.212	11.880	0.001	1.236	1.096–1.394
Dampness syndrome(No = 0,Yes = 1)	0.696	4.336	0.037	2.006	1.042–3.864
*APOA5* rs2075291 genotype(GG = 0,GT = 1)	1.312	5.083	0.024	3.715	1.187–11.628
*CIDEB* rs2144492 genotype (GG = 0,TG = 1,TT = 2)	0.683	3.838	0.05	1.980	1.000–3.920
Constant	−7.671	21.792	0.000	0.001	

## Discussion

This study revealed that the genotype frequencies of *APOA5 rs2075291* and *CIDEB rs2144492* in the overall HTG group differed significantly from those in the control group. The pathogenesis of hypertriglyceridemia is influenced by both genetic and environmental factors. Single nucleotide polymorphism, the most prevalent form of genetic variation among individuals, refers to DNA sequence variations where a single nucleotide in a gene (or genome) differs among members of a biological species or within an individual’s paired chromosomes (Garelnabi et al., 2013). Previous studies have shown that secondary alleles of several common SNPs at the human *ApoA5* gene locus are significantly associated with elevated plasma TG levels (Pennacchio et al., 2002; Kluger et al., 2008). Notably, the *ApoA5 rs2075291* polymorphism has been found to be closely linked to TG levels in the Chinese population, but not in Caucasians (Kao et al., 2003; Hubácek et al., 2004). *CIDEB* influences gene expression across multiple metabolic pathways and signaling networks, including lipid droplet formation, adipogenesis, glycolysis, and gluconeogenesis (Xu et al., 2016; Gong et al., 2009; Chen et al., 2020). For instance, overexpression of *CIDEB* in goat mammary epithelial cells (GMECs) significantly upregulates genes involved in fatty acid synthesis, lipid droplet formation, and triacylglycerol (TAG) synthesis (He et al., 2024). Our findings confirm that polymorphisms at the *APOA5 rs2075291* and *CIDEB rs2144492* loci are associated with hypertriglyceridemia.

Previous studies have reported the impact of lipid regulators on SNPs. For example, interactions between dietary factors and SNPs within the *ApoA1*/*ApoC3*/*ApoA4*/*ApoA5* gene cluster have been documented (Chen et al., 2009; Chien et al., 2009), suggesting that external factors may modulate the expression of gene polymorphisms. Dampness syndrome in traditional Chinese medicine represents a syndrome state developed in specific environmental contexts. According to TCM theory, this syndrome arises from both internal and external dampness pathogens. Earlier research (Zhang Bc, 2020) has shown that TG levels in patients with phlegm-dampness hyperlipidemia are significantly higher than those in other constitution groups. Our prior study (Zhou et al., 2024) further revealed a positive correlation between serum TG levels and the severity of dampness syndrome. Notably, the relationship between TCM dampness syndrome and gene polymorphisms and their combined effect on hypertriglyceridemia has not been previously reported.

This study investigates the association between *APOA5 rs2075291* and *CIDEB rs2144492* polymorphisms and HTG in a dampness syndrome population. Key findings include: in the dampness syndrome subgroup, the HTG group showed a higher count of *CIDEB rs2144492* TG/TT genotypes than the control group, though the difference was not statistically significant (*p* = 0.146). Before FDR correction, the comparison of allele frequencies between the two groups showed a significant intergroup difference (*p* < 0.05), suggesting that *CIDEB rs2144492* variants may enhance HTG susceptibility in individuals with dampness syndrome. This association was not observed in the non-dampness subgroup, implying a potential interaction between dampness syndrome and this gene locus in HTG pathogenesis. Correlation analysis revealed differential associations of HTG with *APOA5 rs2075291* genotype between dampness and non-dampness groups. Multivariate logistic regression identified male gender, higher BMI, dampness syndrome, and *APOA5 rs2075291* genotype as independent risk factors for HTG (*p* < 0.05), while *CIDEB rs2144492* genotype trended toward significance (*p* = 0.05). Potential explanations for these findings include: 1. The associations of *APOA5 rs2075291* or *CIDEB rs2144492* with HTG may be modulated by TCM dampness syndrome; 2. The relatively small sample size may have limited the study’s statistical power to detect subtle associations; 3. HTG is a complex trait influenced by multiple genes (Steinhagen-Thiessen et al., 2017; Kypreos and Zannis, 2006; Guardiola and Ribalta, 2017; Xu et al., 2012) and environmental factors, contributing to heterogeneity in gene–disease associations.

This study reports for the first time the association between *APOA5 rs2075291* and *CIDEB rs2144492* polymorphisms and hypertriglyceridemia in a dampness syndrome population, offering critical insights for investigating triglyceride metabolism in TCM dampness syndrome cohorts. Baseline characteristic analysis showed significant group differences in gender distribution, BMI, and waist circumference between the observation and control groups. To minimize confounding by these baseline factors, gender and BMI were included as covariates in the multivariate logistic regression model. After covariate adjustment, the associations between *APOA5* gene polymorphism, dampness syndrome, and HTG remained statistically significant.

### Limitations

This study recruited participants from outpatient clinics, which may introduce selection bias. For example, outpatients differ from the general population in disease severity, treatment compliance, and help-seeking behaviors, potentially biasing the observed associations between *APOA5/CIDEB* polymorphisms and HTG. Additionally, the single-center outpatient sample limits external validity to populations with similar healthcare-seeking patterns and clinical profiles, hindering generalizability to non-visited or geographically distinct groups.

Post-hoc power analysis using expected MAF and observed ORs showed that with n = 200, the power was only ∼51% for *APOA5 rs2075291* (MAF = 0.1, OR = 2.241) and ∼47.2% for *CIDEB rs2144492* (MAF = 0.19, OR = 1.8)-both far below the 80% statistical benchmark. This indicates insufficient precision in effect estimation. For low-frequency variant association studies, small sample sizes may miss true effects or yield non-reproducible results due to random error. Study designs should pre-calculate sample size based on MAF and target OR to avoid unreliable conclusions. Future research could integrate community epidemiological surveys to comprehensively assess genotype-TCM dampness syndrome interactions in general populations.

## Conclusion

This study confirms that *APOA5 rs2075291* and *CIDEB rs2144492* polymorphisms are associated with hypertriglyceridemia. Individuals with dampness syndrome carrying *CIDEB rs2144492* variants may have an increased predisposition to HTG. These findings advance our understanding of the genetic underpinnings of HTG and may inform the development of personalized preventive strategies for at-risk populations.

## Data Availability

The original contributions presented in the study are publicly available. This data can be found here: https://doi.org/10.17632/pcpg6pcs7f.1.
